# Unraveling Determinants of Affinity Enhancement in Dimeric Aptamers for a Dimeric Protein

**DOI:** 10.1038/s41598-019-54005-4

**Published:** 2019-11-28

**Authors:** Sepehr Manochehry, Erin M. McConnell, Yingfu Li

**Affiliations:** 1 Department of Biochemistry and Biomedical Sciences, McMaster University, 1280 Main St. W., Hamilton, ON L8S 4K1 Canada; 2 Department of Chemistry and Chemical Biology, McMaster University, 1280 Main St. W., Hamilton, ON L8S 4K1 Canada

**Keywords:** Biochemistry, Chemical biology

## Abstract

High-affinity aptamers can be derived *de novo* by using stringent conditions in SELEX (Systematic Evolution of Ligands by EXponential enrichment) experiments or can be engineered post SELEX via dimerization of selected aptamers. Using electrophoretic mobility shift assays, we studied a series of heterodimeric and homodimeric aptamers, constructed from two DNA aptamers with distinct primary sequences and secondary structures, previously isolated for VEGF-165, a homodimeric protein. We investigated four factors envisaged to impact the affinity of a dimeric aptamer to a dimeric protein: (1) length of the linker between two aptamer domains, (2) linking orientation, (3) binding-site compatibility of two component aptamers in a heterodimeric aptamer, and (4) steric acceptability of the two identical aptamers in a homodimeric aptamer. All heterodimeric aptamers for VEGF-165 were found to exhibit monomeric aptamer-like affinity and the lack of affinity enhancement was attributed to binding-site overlap by the constituent aptamers. The best homodimeric aptamer showed 2.8-fold better affinity than its monomeric unit (*K*_d_ = 13.6 ± 2.7 nM compared to 37.9 ± 14 nM), however the barrier to further affinity enhancement was ascribed to steric interference of the constituent aptamers. Our findings point to the need to consider the issues of binding-site compatibility and spatial requirement of aptamers for the development of dimeric aptamers capable of bivalent recognition. Thus, determinants highlighted herein should be assessed in future multimerization efforts.

## Introduction

Multivalent interactions are ubiquitous in nature^1^. For example, DNA binding sites for transcription factors can occur in clusters, which are then bound by oligomeric transcription factors during transcriptional control^2^. Motivated by the observed affinity enhancements associated with multivalency in natural systems^3^, bioengineers have been pursuing synthetic multivalency systems to recognize a protein target. These efforts have led to the development of multivalent forms of antibodies^4,5^ and nucleic acid aptamers^6,7^.

Using a dimer to recognize a protein target represents the simplest multivalency system. There are two types of dimeric recognition systems, a heterodimer comprised of two different recognition elements and a homodimer made of two identical binders. Heterodimeric systems can be applied to any protein target, but they must be engineered from two different recognition elements that each recognize a distinct domain of the same target. Homodimeric systems, on the other hand, can be engineered from a single binder; however, this system only works for a homodimeric protein or a protein containing two or more identical structural domains. Nevertheless, there are many important homodimeric proteins found in biology.

Nucleic acid aptamers are especially suited for multivalency as their selection conditions are easily controlled, they are easily chemically modified^8,9^, and compared to antibodies they are relatively stable and simple to produce^10,11^. There has been a considerable amount of work on engineering dimeric aptamers with varying degrees of success in affinity enhancement (see Supplementary Tables S1 and S2). A few studies have produced dimeric aptamers with substantial (>10-fold) affinity enhancement^6,12,13^. However, many other studies have achieved either modest (~2-fold) affinity improvement^14–18^ or no affinity increase at all^14,19–23^. These results beg the question of what are underlying factors that impact the affinity enhancement when constructing a dimeric aptamer. Previous dimeric aptamer studies have focused almost exclusively on creating optimized linker sequences (the linker issue) that link two component aptamers. Given the fact that this approach does not always create high-affinity dimeric aptamers, other factors must also play important roles. The purpose of the current study is to examine some potentially important factors as discussed below.

The construction of a heterodimeric aptamer for a protein target in general requires at least two different aptamers, which comes with several issues to consider. Alongside the linker issue, the orientation of one aptamer to the other aptamer can be an issue (the orientation issue). In addition, another important condition is that the two aptamers must recognize the same protein target at different sites (binding-site compatibility issue). Furthermore, because aptamers are not small molecules, their significant spatial requirement can impose steric hindrance that prevents non-interfering binding of two aptamers (steric acceptability issue). The construction of a homodimeric aptamer for a homodimeric protein also comes with the linker and steric acceptability issues.

In this study, we carried out a comprehensive investigation examining the feasibility of creating high-affinity dimeric aptamers using three different DNA aptamers previously reported for human vascular endothelial growth factor 165 (VEGF-165)^24–30^. In addition to the availability of three different aptamers, VEGF is a homodimeric protein molecule^31–37^, offering a great opportunity for engineering both heterodimeric and homodimeric aptamers for the same system. Moreover, unlike the human thrombin-DNA aptamer system^38–45^ that has been the subject of many previous heterodimeric aptamer engineering efforts^6,13,17,19,22,46–52^ (see Supplementary Table S2), for aptamer/VEGF-165 systems, no high-resolution structural data are available. For this reason, lessons learned from such a system can serve as generalizable guiding principles for other protein-aptamer systems.

## Results

### Affinity assessment of three monomeric aptamers

We selected the technique of electrophoretic mobility shift assays (EMSA) to assess aptamer binding to VEGF-165 simply because this technique allows for direct observation of the protein-aptamer complex^53^.

**Figure 1:**
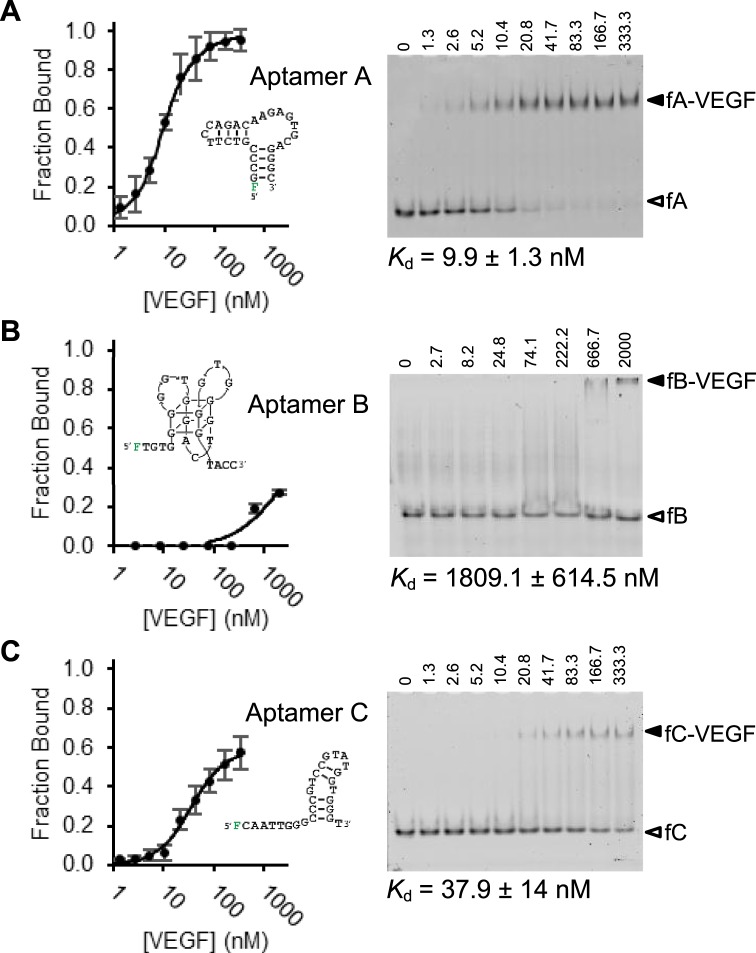
EMSA assays to examine VEGF binding by aptamers A, B and C. [Aptamer] = 2.5 nM for each panel; [VEGF-165] = 0, 1.3, 2.6, 5.2, 10.4, 20.8, 41.7, 83.3, 166.7, 333.3 nM for panel A and C, with four replicates used to generate the binding curve; [VEGF-165] = 0, 2.7, 8.2, 24.8, 74.1, 222.2, 666.7, 2000 nM for panel B. Aptamer:VEGF is ~1:1 in lane 3 for panels A and C, and in lane 2 for panel B. Image for each panel: a representative gel image from EMSA. fA, fB, fC refer to fluorescently labelled aptamers A, B and C. F refers to fluorescein label. Further fX-VEGF represents the aptamer-VEGF complex, where X is either A, B, or C.

There are three distinct classes of VEGF-165 binding DNA aptamers, which were originally isolated from three separate selections^24,25,28^, and further optimized in later studies^26,27,29^. Their sequences and original names are shown in Supplementary Table S3. In this study, they are referred as A, B and C for simplicity, which correspond to the aptamers named +5′GC + 3′C, 3R02, and SL2-B, respectively. Their secondary structures, proposed in respective studies, are given in Fig. 1. The structure of aptamer A is shown as proposed by Potty *et al*.^27^, aptamer B by Nonaka *et al*.^24,29^, and aptamer C by Kaur *et al*.^26^. The complex formation of each aptamer was investigated using a fixed aptamer concentration (2.5 nM) while the concentration of VEGF-165 was increased. The EMSA data provided in Fig. 1 clearly shows that aptamers A and C exhibit significantly higher affinity than aptamer B. The binding affinities (*K*_d_ values) of aptamers A and C are 9.9 ± 1.3 nM and 37.9 ± 14 nM, respectively. Aptamer B, on the other hand, exhibited significantly reduced affinity, with an estimated *K*_d_ in excess of 1.5 μM, despite our substantial efforts to optimize EMSA conditions (see Supplementary Figure S1). Due to its poor affinity, aptamer B was not further investigated.

### Assessment of compatibility of aptamers A and C for constructing a heterodimeric aptamer

Aptamers A and C were chosen to construct a heterodimeric aptamer given their strong affinity to VEGF-165 and their differences in primary sequences and secondary structures. However, a critical issue to address is their ability to simultaneously bind the same protein molecule. It should be noted that VEGF has different isoforms^31–37^, and the isoform we used up to this point was VEGF-165, which has two distinct domains known as the HBD (heparin-binding domain) and the RBD (receptor-binding domain). Aptamer C has been reported to bind the HBD^26^, based on the finding that it does not bind VEGF-121, a smaller isoform lacking the HBD. This approach was used, rather than investigating the HBD fragment alone, to help deduce the domain to which the aptamer binds. Such information was not available for aptamer A.

We performed EMSA with aptamer A and VEGF-121. No complex formation was observed even at high protein concentrations (Supplementary Figure S2). This result, along with the fact that aptamer A strongly binds VEGF-165, indicated that aptamer A also binds the HBD, a property shared by aptamer C.

**Figure 2:**
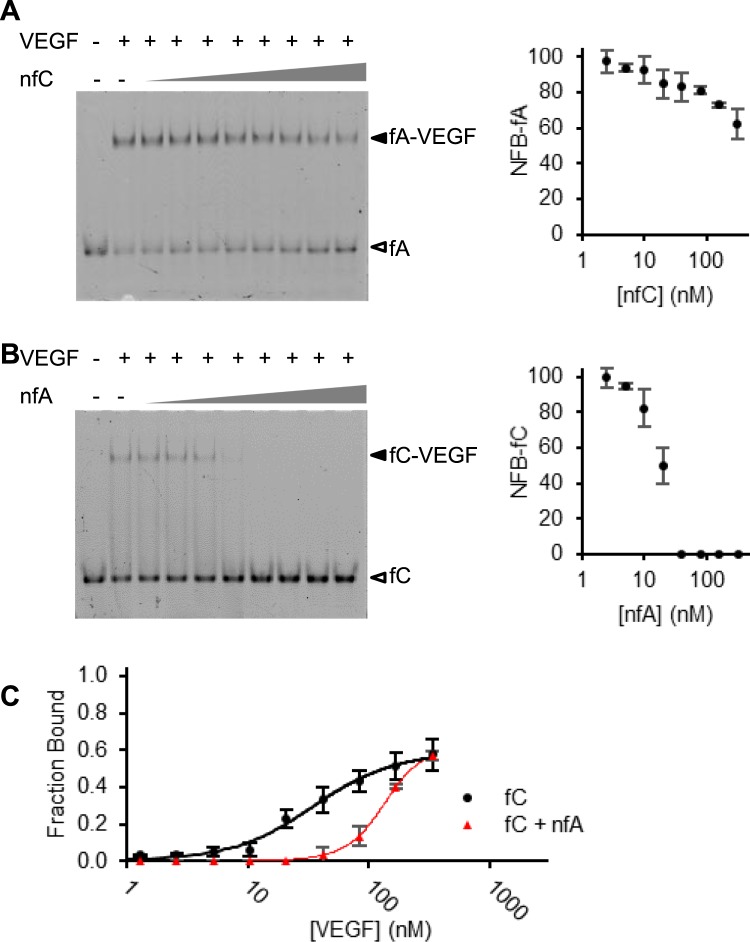
Aptamers A and C competition assays. (**A**) Binding of VEGF-165 (10 nM) by fluorescent aptamer A (fA; 2.5 nM) competed by non-fluorescent aptamer C (nfC) at 0, 2.5, 5, 10, 20, 40, 80, 160, 320 nM (lanes 2–10). (**B**) Binding of VEGF-165 (50 nM) by fC (2.5 nM) competed by nfA at 0, 2.5, 5, 10, 20, 40, 80, 160, 320 nM (lanes 2–10). Each panel shows a representative gel image (left) and the graph of NFB as a function of non-fluorescent competitor concentration (right). (**C**) Fraction of VEGF-165 bound fC when 2.5 nM fC (black circles) or 2.5 nM nfC + 25 nM nfA (red triangles) were mixed with 1.3, 2.6, 5.2, 10.4, 20.8, 41.7, 83.3, 166.7, and 333.3 nM VEGF-165.

However, it remained possible that aptamers A and C could still simultaneously bind VEGF-165 if their binding sites do not overlap. To assess this possibility, we performed EMSAs to analyze the competition for binding to VEGF-165 either by fluorescent aptamer A (fA; 2.5 nM) and nonfluorescent aptamer C (nfC; varied between 0-320 nM; Fig. 2A) or by fluorescent aptamer C (fC; 2.5 nM) and nonfluorescent aptamer A (nfA; varied between 0–320 nM; Fig. 2B). Note that 10 and 50 nM VEGF-165 were used for the fA/nfC and fC/nfA competitions, respectively, taking into consideration the individual aptamer’s binding affinity differences. To simplify the comparison, we calculated the values of normalized fraction of bound (NFB) of fA or fC using the following equation: NFB = FB/FB_0_ × 100, where FB_0_ and FB represent fraction of a fluorescent aptamer bound in the absence and presence of the competitor, respectively. The relevant NFB values are plotted in the right panels of Fig. 2A,B.

The competition assays depicted in Fig. 2 clearly show that the fraction of bound fA or fC was inversely proportional to the increasing amounts of the non-fluorescent competitor. For example, fC was completely displaced by nfA when the concentration of nfA reached 40 nM (Fig. 2B; note that [fC] = 2.5 nM). These results demonstrate the inability of aptamers A and C to engage with VEGF-165 simultaneously, indicating that they share the same binding site or bind nearby sites that interfere with each other’s binding.

The inability of aptamers A and C to bind VEGF-165 simultaneously was further demonstrated by the data in Fig. 2C which compares the binding curves of fC alone, and fC in the presence of 25 nM nfA as a competitor. The significantly reduced binding affinity of fC in the presence of 25 nM nfA as a competitor (with an apparent *K*_d_ shifted from 37.9 nM to 135.4 nM) is consistent with the incompatibility of simultaneous binding to VEGF-165 by aptamers A and C.

### Assessment of binding to VEGF-165 by heterodimeric aptamers constructed with aptamers A and C

**Figure 3:**
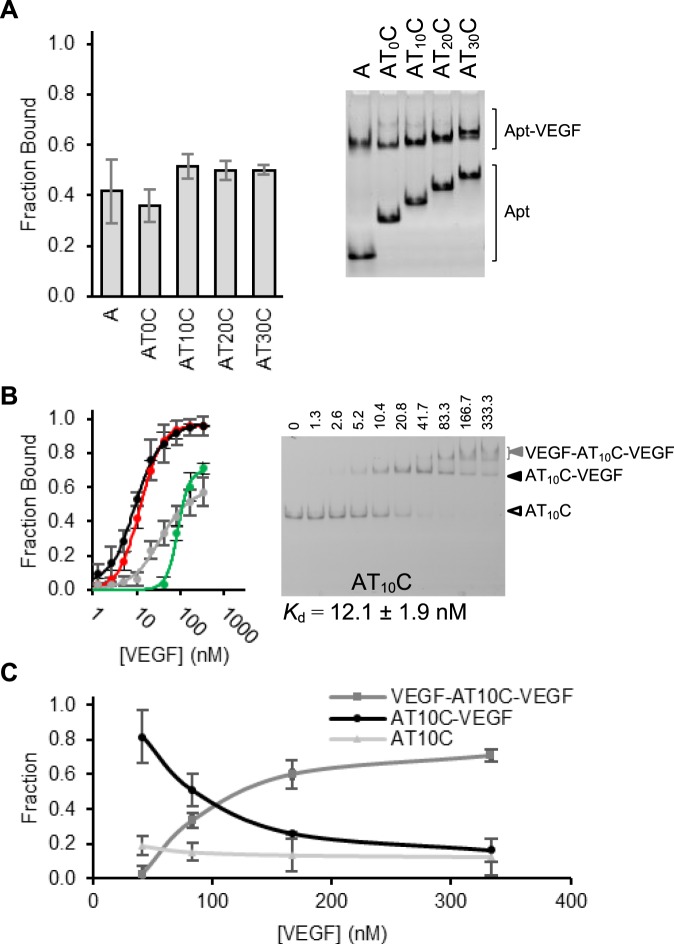
EMSA with A-C heterodimeric aptamers. (**A**) Fraction of fluorescent aptamers (2.5 nM) bound with VEGF-165 (10 nM). Aptamer A (lane 1) and four AT_n_C aptamers were compared in this experiment (n = 0, 10, 20, and 30) (lanes 2–5). Image shows only a portion of the gel. (**B**) Binding curves (left panel) for aptamer A (black), aptamer C (grey), and AT_10_C (red). The curve in red is derived using combined fractions of fluorescent aptamers in both the top DNA band and the middle DNA band, whereas the curve in green is obtained using the fraction of fluorescent aptamer in the top DNA band. The right panel shows a representative EMSA result for AT_10_C binding with VEGF-165 at 0, 1.3, 2.6, 5.2, 10.4, 20.8, 41.7, 83.3, 166.7, 333.3 nM. (**C**) Quantification of three bands indicated by the arrows, carried out for lanes 7–10 (41.7, 83.3, 166.7, 333.3 nM) of EMSA results, where higher-order associations are visible. Fraction indicates the amount of each component, comparing both unbound and bound forms.

The data presented above suggested that aptamers A and C were ill-suited for constructing a heterodimeric aptamer with bivalent recognition. To experimentally verify this point, we created several A-C or C-A dimeric aptamers with and without a poly-thymidine linker. Figure 3A provides the EMSA results obtained with four AT_n_C dimers with n = 0, 10, 20 and 30. The data shows that each AT_n_C dimer was still functional, as reflected by the observation of the second band with significantly reduced gel mobility. In addition, the fraction of the aptamer bound with the protein was not significantly altered by the length of the linker, although the fraction bound seemed to be slightly higher with a T-linker. Similar results were obtained with four CT_n_A heterodimers (Supplementary Fig. S3; n = 0, 10, 20 and 30). The data indicate that the relative A-to-C orientation does not affect the binding affinity in this case. Based on the above results, AT_10_C was chosen for further analysis.

Thus, we carried out an experiment to determine the fraction of AT_10_C bound with VEGF-165 when the protein concentration was increased (Fig. 3B). We made an interesting observation: at the lower VEGF-165 concentrations, a single DNA band with a reduced gel mobility (denoted here as “the middle DNA band” and, marked by a black arrowhead in the gel image in Fig. 3B); at higher VEGF-165 concentrations, however, another DNA band with even smaller gel mobility (the top DNA band, marked by a grey arrowhead) became visible. Given the fact that AT_10_C is a dimeric aptamer, the middle DNA band should be AT_10_C-VEGF binary complex (the 1:1 protein/aptamer complex) while the top DNA band should correspond to VEGF-AT_10_C-VEGF ternary complex (the complex of 2 proteins and 1 aptamer). Consistent with this notion is the observation that the progressive reduction of the fraction of the middle DNA band is accompanied by the gradual increase of the fraction of the top DNA band when the VEGF concentration increased (Fig. 3C).

We also performed further quantitative analysis using EMSA. Two binding curves were established: the first one (shown in red in Fig. 3B) was derived using the combined fractions of aptamers in both the middle and top DNA bands, and the second only accounted for the fraction of the aptamer in the top DNA band (shown in green). The first binding curve overlaps very well with that of monomeric aptamer A; the apparent *K*_d_ derived using this curve is 12.1 ± 1.9 nM, which is nearly identical to that of the monomeric aptamer A (9.9 ± 1.3 nM). This finding strongly suggests that the first binding curve was a result of VEGF-165 binding to the A aptamer domain of AT_10_C. The apparent *K*_d_ derived using the second binding curve is 87.3 ± 4.9 nM, which, as expected, lies in between the *K*_d_ of the monomeric aptamer C alone (37.9 ± 14 nM; Fig. 1C) and the *K*_d_ of the monomeric aptamer C in the presence of 25 nM aptamer A (135.4 ± 2.4 nM; Fig. 2C; note that the concentration of AT_10_C used for Fig. 3B was only 2.5 nM). The binding data comparison is consistent with the binding of second VEGF-165 to the C aptamer domain in the AT_10_C-VEGF complex.

All the observations made with AT_10_C above clearly indicate that AT_10_C contains two functional aptamer elements that cannot simultaneously bind the same VEGF molecule but are capable of binding two different VEGF-165 molecules. These results further confirm the finding made with the aforementioned aptamer displacement assay: aptamers A and C cannot simultaneously bind to one VEGF-165 molecule.

To make sure that the results obtained with AT_10_C was not a result of a small linker, we also synthesized a heterodimeric aptamer with a very long, T_60_ linker (AT_60_C) and obtained its binding curve (Supplementary Figure S4A). These two dimeric aptamers exhibited superimposed binding curves, confirming that the size of the linker was not an issue.

### Assessment of binding to VEGF-165 by homodimeric aptamers

The confirmed high affinity of aptamers A and C served as a good start point for constructing homodimeric aptamers for VEGF-165, given that this protein is composed of two symmetrical monomers.

We constructed four AT_n_A and four CT_n_C aptamers once again choosing a poly-T linker of 0, 10, 20, and 30 nucleotides. We then made side-by-side comparisons in a single EMSA assay in which we determined the fraction of fluorescently labelled aptamer bound with VEGF-165 at relatively low aptamer and VEGF concentrations ([aptamer] = 2.5 nM; [VEGF-165] = 10 nM).

**Figure 4:**
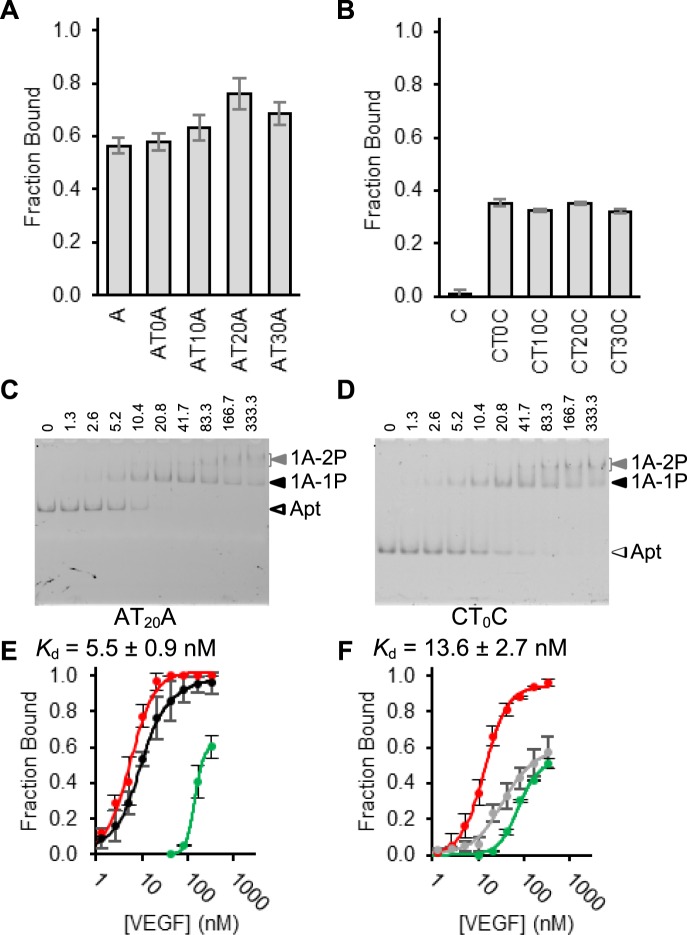
Assessment of binding of homodimeric aptamers A and C. Comparative binding of (**A**) aptamer A and four AT_n_A homodimers, and (**B**) aptamer C and four CT_n_C homodimers. Fraction of each fluorescent aptamer (2.5 nM) bound with VEGF-165 (10 nM) was shown. (**C,D**) Representative gel image for (**C**) 2.5 nM AT_20_A and (**D**) 2.5 nM CT_0_C in the presence of 0, 1.3, 2.6, 5.2, 10.4, 20.8, 41.7, 83.3, 166.7, 333.3 nM VEGF-165. (**E**) Binding curves for aptamer A (black) and AT_20_A (red). (**F**) Binding curves for aptamer C (grey) and CT_0_C (red). For both E and F, the curve in red is derived using combined fractions of fluorescent aptamers in both the top DNA band and the middle DNA band, whereas the curve in green is obtained using the fraction of fluorescent aptamer in the top DNA band.

For the AT_n_A series, AT_20_A exhibited the best binding and its fraction bound was considerably higher than that of the monomeric aptamer A (0.76 vs. 0.57; Fig. 4A). For the CT_n_C series, the fraction bound by monomeric aptamer was only 0.01 (the first bar in Fig. 4B); however, the fraction bound by all four homodimeric aptamers was found to be ~0.35 (Fig. 4B), representing substantial increases.

We then performed the titration experiment with 2.5 nM AT_20_A (Fig. 4C) and CT_0_C (Fig. 4D) when VEGF-165 was used at 0, 1.3, 2.6, 5.2, 10.4, 20.8, 41.7, 83.3, 166.7, and 333.3 nM. Similar to the case with the heterodimeric aptamer AT_10_C, we also observed the middle DNA band and the top DNA band for both homodimeric aptamers and found that the amount of the top DNA band gradually increased whereas that of the middle DNA band reduced with higher VEGF-165 concentrations. The middle and top DNA bands were labelled as 1A-1P and 1A-2P, respectively, as we believe they corresponded to the 1:1 and 1:2 aptamer/protein complexes.

To confirm the top DNA band indeed represented the 1:2 aptamer/protein complex, we created AT_20_A_m_ in which the second aptamer was inactivated by base mutations (see Supplementary Figure S5). When AT_20_A_m_ was examined by EMSA, the top DNA band was absent (Supplementary Figure S5).

The *K*_d_ values were then determined for AT_20_A (Fig. 4C) and CT_0_C (Fig. 4D). Two binding curves were established, one with the combined fractions of fluorescent aptamers in both the middle and top DNA bands (red curves), one with only the fraction of the aptamer in the top DNA band (green curves). The *K*_d_ derived with the red curve of AT_20_A was 5.5 ± 0.9 nM, 1.8-fold better than the monomeric aptamer A (*K*_d_ = 9.9 ± 1.3 nM). The equivalent *K*_d_ for CT_0_C was 13.6 ± 2.7 nM, 2.8-fold better than monomeric aptamer C (*K*_d_ = 37.9 ± 14 nM). The apparent *K*_d_ values derived using the green curve of AT_20_A and CT_0_C is 145 ± 11.6 nM and 74.8 ± 3.5 nM, respectively, indicating the 1:2 aptamer/VEGF-165 complex for each dimeric aptamer can only occur at significantly higher VEGF-165 concentrations than that required for the 1:1 complex.

Furthermore, our assessment of AT_60_A (*K*_d_ = 7.0 ± 1.5 nM; Supplementary Figure S4B) and CT_60_C (*K*_d_ = 22.4 ± 1.0 nM; Supplementary Figure S4C) eliminated concerns regarding the length of the spacer as a limiting factor, as both homodimers demonstrated affinity similar to their smaller counterparts.

The binding affinity analysis above reveals that the homodimeric aptamers, AT_20_A and CT_0_C, behaved considerably different from the heterodimeric aptamer AT_10_C featured earlier. While no affinity improvement was detected with the heterodimeric aptamer, both homodimeric aptamers produced notable affinity enhancement. The lack of affinity improvement in AT_10_C was attributed to the inability of both aptamers to simultaneously bind to the same target. It was therefore interesting to determine if VEGF-165 could simultaneously accommodate two A aptamers or two C aptamers.

Experimentally testing the above idea represents a challenge for a particular reason: the gel mobility of the aptamer-VEGF complex was mostly dictated by VEGF-165 and to a much less degree by the size of the aptamer. This was clear from the experiment performed in Fig. 3A where the VEGF-165 complex with monomeric A aptamer and all four heterodimeric aptamers (AT_0_C, AT_10_C, AT_20_C and AT_30_C) had similar gel mobility. For example, the VEGF-165 complex with A and AT_10_C had nearly identical gel mobility (Fig. 3A), even though the monomeric aptamer A contains only 28 nucleotides whereas AT_10_C has 64 nucleotides.

However, we did observe detectable differences in gel mobility between the monomeric aptamer A and the heterodimeric aptamer AT_30_C (Fig. 3A). Based on this observation, we first created nfAT_100_ and nfCT_100_, nonfluorescent aptamer A and C tagged with 100 T residues on their 3′ end. We then used them to examine co-binding with fA or fC (FAM-labelled A and C), as described below.

**Figure 5:**
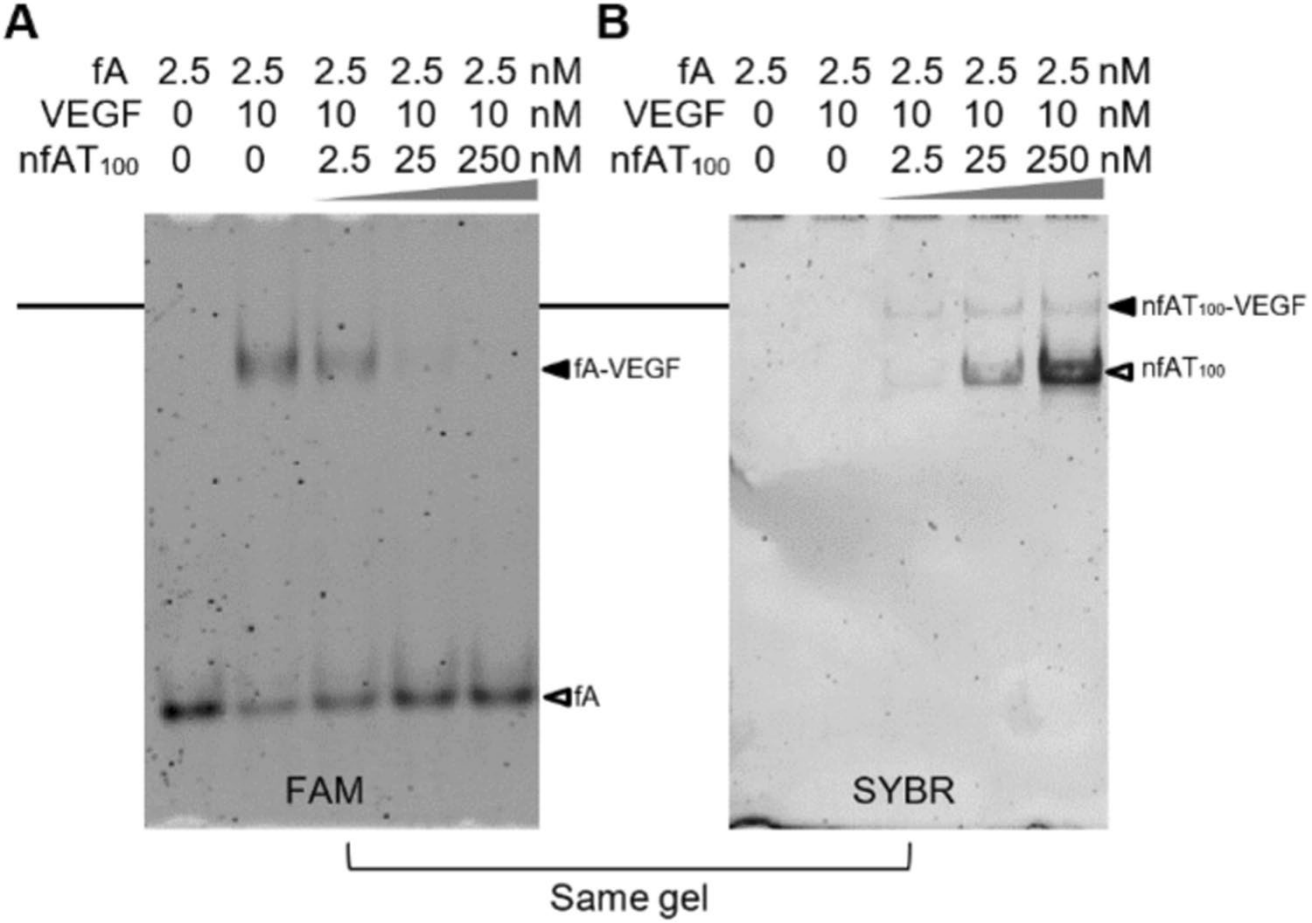
fA and nfAT_100_ competition assays. **(A)** Fluorescent gel image of 2.5 nM fA alone (lane 1) and 2.5 nM fA in the presence of 10 nM VEGF-165 (lanes 2–5) as well as 2.5, 25, 250 nM (lanes 3–5, respectively) nfAT_100_. **(B)** SYBR gold stained version of same gel.

Figure 5A is a fA-tracking gel image from the EMSA experiment conducted with 2.5 nM fA and 10 nM VEGF-165 in the presence of 2.5, 25 and 250 nM nfAT_100_. This image allows for identification of fA and its complexes with VEGF; however, in this image nfAT_100_ would not be detected. Figure 5B is the image of the same gel obtained after it was stained by SYBR gold. This image can reveal the location of nfAT_100_ and its complex with VEGF; however, fA was not detected in this image due to its small size and low concentration.

The purpose of the above experiment was to determine if the DNA band that corresponds to fA-VEGF-nfAT_100_ could be detected in the image of Fig. 5A, which should appear at the location of black line, when fA and nfAT_100_ were mixed with VEGF-165. No such band was observed when nfAT_100_ was tested at 2.5, 25 and 250 nM. Instead, when the concentration of nfAT_100_ was increased, growing amounts of fA was displaced from the fA-VEGF complex (Fig. 5A). Similar results were obtained with fC and nfCT_100_ (Supplementary Figure S6). Collectively, these results strongly suggest that one VEGF-165 molecule cannot simultaneously accommodate two aptamer A molecules or two aptamer C molecules.

## Discussion

Given that EMSA represents a very simple method to visualize the binding between a labelled aptamer and its protein target, and at the same time gather useful size and stoichiometry information, it was chosen in this study to assess the binding behaviours of 3 monomeric and 23 dimeric VEGF-165 binding DNA aptamers.

There are three distinct classes of existing monomeric VEGF-binding DNA aptamers, which are renamed as aptamers A, B and C in this report (Fig. 1). We were able to show using EMSA that aptamers A and C are high-affinity binders (with nanomolar *K*_d_) while aptamer C has significantly reduced affinity (with micromolar *K*_d_). Given this observation, we focused our subsequent dimeric aptamer work on aptamers A and C.

We were also able to use this method to assess whether VEGF-165 can simultaneously bind aptamers A and C (Fig. 2). This was done using a competition assay employing a fluorescently labelled aptamer as the tracer and an unlabelled aptamer as the competitor (Fig. 3). This led to the finding that these two aptamers cannot be simultaneously accommodated by the same VEGF-165 molecule. Expectedly, all 9 dimeric aptamers constructed with A and C bind VEGF-165 with the affinity observed for the monomeric aptamer A alone (Fig. 3), the better aptamer of the two. Taken together, our results indicate that aptamers A and C have overlapping binding sites, making them ill-suited for development of high-affinity heterodimers. These findings further signify that differences in primary sequences and secondary structures between two aptamers do not necessarily translate into fruitful dimeric aptamers. However, in the absence of structural and binding site information, a quick way to assess the suitability of an aptamer pair for building a high-affinity dimeric aptamer is to conduct an EMSA based displacement assay.

For a homodimeric protein such as VEGF-165, in theory it should be possible to set up a bivalent aptamer from a single aptamer. This can be done through the use of a DNA molecule containing two identical aptamer domains joined to each other via a linker domain. Five homodimeric A aptamers and 5 homodimeric C aptamers were constructed using this strategy and they all showed an enhanced activity over their monomeric counterparts, with the best construct, CT_0_C, exhibiting 2.8-fold enhancement.

However, the affinity enhancement observed with these homodimeric aptamers is still moderate. This made us wonder whether one VEGF-165 molecule can simultaneously accommodate two aptamer A or C molecules. To investigate this, we used a competition assay much alike the one employed for heterodimer analysis. To make this experiment possible, we set up the competition between the fluorescently labelled aptamer A (fA) and the non-fluorescent aptamer A tagged with 100 thymidines (AT_100_). This design creates enough gel mobility difference to detect the 1:1 and 2:1 aptamer/VEGF-165 complexes. The latter complex was not observed at all; instead, the amount of the fA-VEGF complex was reduced in the presence of increasing amounts of AT_100_ (Fig. 5). The same experiment performed for aptamer C produced nearly identical results. These two experiments clearly demonstrate that one VEGF-165 molecule cannot simultaneously accommodate two A or C aptamer molecules. The results seem to suggest that aptamer A (and C) binds the dimeric VEGF-165 with an orientation that spatially clashes with the second aptamer. These results also point to the necessity of experimentally examining the ability of a homodimeric protein to accommodate two identical aptamers.

One observation worth commenting on is that the two aptamer domains in the featured heterodimeric and homodimeric aptamers are functional, indicated by the presence of the 1:2 aptamer/VEGF-165 complexes at high protein concentrations. This finding rules out the possibility of functional interference by putting two aptamer domains in one DNA sequence.

Additionally, the lack of simultaneous binding by the dimeric aptamers to the dimeric VEGF protein is not due to the inadequate linker length in the dimeric aptamers, based on the observation that dimeric aptamers with a linker region of varying numbers of thymidines (up to 60) exhibited similar binding activities. We investigated one alternate orientation of the component aptamers (i.e. ATnC vs CTnA) and showed no notable changes in binding affinity, however there exist several other approaches to achieving alternate arrangement of component aptamers. This includes the use of more flexible linkers, such as polyethylene glycol linkers, or 5′-5′ dimeric aptamer arrangement.

Previous studies have shown that aptamer C is an HBD-specific aptamer^26^ and aptamer B an RBD-specific aptamer^29^; our current work here has revealed that aptamer A also binds HBD but not RBD. No study has made head-to-head comparison to determine the relative binding affinity of these three aptamers until our current study. Using EMSA, we showed that both aptamers A and C exhibited much higher affinity to VEGF-165 (nM-*K*_d_) than aptamer B (μM-*K*_d_). Upon the conclusive finding that aptamers A and C were ill-suited for constructing a bivalent aptamer, we did make an attempt to build dimeric A/B or B/C aptamers. Unfortunately, when A/B and B/C aptamers were combined into a single DNA molecule, the resultant aptamers did not show any improved binding to VEGF-165 over the A aptamer alone (Supplementary Figure S7). We believe the significantly reduced affinity is the key reason for this observation.

For successful engineering of dimeric aptamers with bivalent interactions with VEGF-165, our findings point to the need for new high-affinity aptamers that meet one of the following three conditions. Firstly, the new aptamers bind VEGF-165 at a site that does not interfere with the binding of aptamer A or C, and such aptamers should be suitable for setting up heterodimeric aptamers. Secondly, we propose a possible solution could be to use aptamers with similar affinity, so that one aptamer does not predominate the binding outcome, thereby enabling both to engage simultaneously. Thirdly, the new aptamers bind VEGF-165 at a suitable region with a spatial orientation that does not interfere with the binding of the second, same, aptamer, and such aptamers should be useful for setting up homodimeric aptamers. Searching for these aptamers, however, may represent a great challenge, given the fact that although two separate SELEX experiments conducted by two different laboratories using two different DNA libraries led to the discovery of two sequence-distinct aptamers, they share overlapping binding sites within the HBD of VEGF-165. It is highly likely that the selection of these two aptamers are linked to the pI value of ~11.3 of the HBD^35^– the high pI of the HBD makes this domain positively charged, significantly favouring the selection of negatively charged aptamers to simply recognize this domain by charge-charge interactions. Therefore, novel strategies are required to drive the enrichment of high-affinity aptamers capable of binding VEGF-165 away from the HBD.

## Conclusion

Although high-affinity protein binding aptamers can be engineered via dimerization of existing aptamers^59–76^, multiple factors have to be carefully considered and examined to maximize the success rate. Although the length of the linker between two aptamer domains and the linking orientation of two constituent aptamers are important elements to evaluate, the two most important criteria to consider are the binding-site compatibility of the two aptamers and their spatial acceptability. The differences in primary sequences and secondary structures do not necessarily mean that they recognize different epitopes of the same protein. Furthermore, the existence of a high-quality aptamer for a dimeric protein does not necessarily guarantee the successful engineering of a homodimeric aptamer with significantly improved affinity over the monomeric aptamer as the spatial requirement of this aptamer may prevent the co-binding of the two aptamer domains in the dimeric aptamer to the same protein molecule. As we continue to pursue higher affinity aptamers, these factors need to be considered early in the process so that effective SELEX protocols can be designed that drive the selection of aptamers that meet these essential requirements.

## Materials and Methods

All fluorescently labelled and unmodified DNA oligonucleotides were purchased from Integrated DNA Technologies (Coralville, IA, USA), and purified by standard 10% denaturing (8 M urea) polyacrylamide gel electrophoresis (dPAGE)^54^. Each oligonucleotide was obtained from IDT along with mass spectrometry data, a representative spectrum is shown in Supplementary Figure S9. Their sequences are provided in Supplementary Table S4. VEGF-121 and VEGF-165 (his-tagged, expressed, purified from HEK-293 cells) were obtained from AcroBio (Newark, DE, USA). All other reagents were purchased from Sigma Aldrich (Oakville, Canada). All reagent solutions were either autoclaved or filtered using 0.2 μm syringe filters. Fluorescence gel images were obtained with Typhoon 9200 (GE, Healthcare, Piscataway, NJ, USA) immediately after gel running. Quantification of DNA bands in the image was performed with ImageQuant (Amersham) software, using the formula Fraction bound = (intensity of bound band)/(bound + unbound). Any partial gels are shown in full in Supplemental Figure S8. All graphs were made using Excel (Microsoft, Redmond, WA, USA), and observed binding curves were used to determine dissociation constants by fitting the data to the quadratic equation using the solver feature of Microsoft Excel^55,56^, as shown previously^57,58^.

### Aptamer binding assays

Each aptamer was used at 2.5 nM if not otherwise specified. A relevant aptamer was heated at 90 °C for 2 minutes, cooled to room temperature, and then incubated with VEGF-165 for 1 hour at defined concentrations described in the relevant figure legends. The total reaction volume of a typical reaction was 20 µL. Two replicates were performed unless otherwise stated. Phosphate buffered saline (PBS with 0.005% v/v Tween 20, pH 7.4) was used as the binding buffer for all aptamers, with the exception for aptamer B (which forms a G-quadruplex structure) for which the PBS buffer also contained 50 mM KCl^24^. At the completion of the binding reaction, a 5× loading dye solution composed of 25% glycerol and bromophenol blue (1 mg/mL) was mixed with each reaction solution, and 10 µL of the resulting mixture was loaded into either a 10- or 15-well 7.5% acrylamide gel with 1.5 mm gel thickness. The gel was made using 37.5: 1 acrylamide: bis-acrylamide and polymerized for 15 minutes. Each gel was run with 0.5× Tris-borate (TB, pH 8.3) buffer at 180 V for 30 minutes inside a 4 °C cold-room. Following gel running, the gel was scanned with the Typhoon at 600 volts with a 200 µm pixel size for FAM fluorescence using the FAM filter configuration (excitation at 494 nm and emission at 525 nm).

### Determination of binding domain for aptamer A

This was performed using the similar protocol described above following a 1-hour incubation of 2.5 nM aptamer A with varying concentrations of VEGF-121 given in the legend of Supplementary Figure S2.

### Heteromeric displacement

This was done with either the fA and nfC pair or the fC and nfA pair using the similar protocol as described above. For the fA and nfC pair, 2.5 nM fA was incubated with 10 nM VEGF-165 in the presence of varying concentrations of nfC given in the legend of Fig. 2A. For the fC and nfA pair, 2.5 nM fA was incubated with 50 nM VEGF-165 in the presence of varying concentrations of nfC given in the legend of Fig. 2B.

### Homomeric displacement

This experiment was done with either the fA and nfAT_100_ pair or the fC and nfCT_100_. For fA and nfA AT_100_ as follows: 2.5 nM fA was incubated with 10 nM VEGF-165 in the presence of 0, 2.5, 25, 250 nM nfAT_100_. And similarly for fC and nfCT_100_ using concentrations given in legend of Supplementary Figure S6. This was followed by EMSA. The gel was then scanned with the Typhoon to obtain the FAM fluorescence. The same gel was then stained with SYBR gold for 20 minutes, then the gel was rescanned using the Typhoon.

### Determining the binding curve of fC in the presence nfA

The experiment was performed with 2.5 nM fC in the presence of 25 nM nfA. 2.5 nM fC was incubated in a 20 µL solution for 30 minutes with varying concentrations of VEGF-165 given in the legend of Fig. 2C. Then 1 µL of 0.525 µM nfA was added, bringing the final nfA concentration to 25 nM, without significantly altering the concentrations of fC and VEGF-165.

## Supplementary information

Supplementary Information
