# Supplementary material for: Unraveling Determinants of Affinity Enhancement in Dimeric Aptamers for a Dimeric Protein
Source: Sci Rep. 2019 Nov 28;9:17824. doi: 10.1038/s41598-019-54005-4 (PMC6883073; doi:10.1038/s41598-019-54005-4)
Supplement: Supplementary file 1 — Supplementary Information [file 41598_2019_54005_MOESM1_ESM.pdf]

Supplementary Information for :

Unraveling Determinants of Affinity Enhancement in Dimeric Aptamers for a Dimeric Protein

Sepehr Manochehry[1], Erin M. McConnell[1], and Yingfu Li[1,2,*]

[1]Department of Biochemistry and Biomedical Sciences, McMaster University, 1280 Main St. W., Hamilton, ON L8S 4K1, Canada.
[2]Department of Chemistry and Chemical Biology, McMaster University, 1280 Main St. W., Hamilton, ON L8S 4K1, Canada.
*Correspondence and requests for materials should be addressed to Y.L. (email: liying@mcmaster.ca)

Supplementary Table S1. Affinity enhancement of homodimeric aptamers. Composition shown as DNA (deoxyribonucleic acids), RNA (ribonucleic acids), 2′-F-RNA (2′-fluoropyrimidine–modified RNA), and LNA (locked nucleic acids).

Protein Target	K_d (nM)	Nucleic acid composition	Ref.
Vitronectin	Monomer: 408 ± 78 nM; Dimer: 485 ± 78 nM<br>**No enhancement**	DNA	[20]
L-selectin	Monomer: 0.7 nM; Dimer: 0.8 nM<br>**No enhancement**	DNA	[23,59,60]
CD28	Monomer: 60 nM; Dimer: 60 nM<br>**No enhancement**	2'-F-RNA	[21]
mIgM (B-cell receptor)	Monomer@4°C: 43 nM; Dimer @4°C: 57 nM<br>**No enhancement**<br><br>Monomer @37°C: >10000 nM; Dimer @37C°C: 6222 nM<br>**<2-fold enhancement**	LNA/DNA	[14]
GFP	Monomer: 5.1 nM; Dimer: 3.2 nM<br>**1.6-fold enhancement**	RNA	[15]
TATA-binding protein	Monomer i (A-37): 25 nM; Dimer i (A-37): 15 nM<br>**1.7-fold enhancement**<br><br>Monomer ii (V-1): 45 nM; Dimer ii (V-1): 25 nM<br>**1.8-fold enhancement**<br><br>Monomer iii (V-2): 60 nM; Dimer iii (V-2): 30 nM<br>**2-fold enhancement**	RNA	[16]
PTK7	Monomer: 0.86 ± 0.21 nM; Dimer: 0.30 ± 0.06 nM<br>**2.9-fold enhancement**	DNA	[18,61–65]
HSF1	Monomer: 32 ± 10 nM; Dimer: 0.5 ± 0.1 nM<br>**64-fold enhancement**	RNA	[12]
CTLA-4	Not assessed	2'-F-RNA	[66]
4-1BB	Not assessed	2'-F-RNA	[67]
CD30	Not assessed	DNA	[68]
OX40	Not assessed	2'-F-RNA	[69]
HTR	Not assessed	2'-F-RNA	[70]
MUC-1	Not assessed	DNA	[71]
CD4	Not assessed	RNA	[72]
LAG3 (CD223)	Not assessed	2'-F-RNA	[73]
DFHBI	Not assessed	RNA	[74]
PSMA	Not assessed	DNA	[75]

Supplementary Table S2. Affinity enhancement in heterodimeric aptamers for thrombin. Thrombin-binding aptamers dimerized and compared to their monomeric forms, with dissociation constants (K_d) provided with standard deviation values where available.

Component	Aptamer	K_d (nM)	Affinity enhancement	**Reference**
Heterodimer<br>Monomer a<br>Monomer b	T_3-Boc- T_{10}-Tas<br>T_3-Boc<br>T_9-Tas	54 ± 4.7<br>60 ± 7.9<br>66 ± 22	No enhancement	22
Heterodimer<br>Monomer a<br>Monomer b	HD1-5dA-60.29<br>HD1<br>60.29-15dA	4.9 ± 1.6<br>50.9.1 ± 6.3<br>4.3 ± 2.0	No enhancement	19
Heterodimer<br>Monomer a<br>Monomer b	4HB-A1-B4<br>4HB-A1<br>4HB-B4	~10<br>20-50<br>>50	~2-5-fold	17
Heterodimer<br>Monomer a<br>Monomer b	16T<br>Bock-15<br>Tasset-29	0.12<br>2.5<br>1.5	12.5-fold	13
Heterodimer<br>Monomer a<br>Monomer b	Linker 5<br>15-mer<br>29-mer	0.14<br>20.2<br>3.5	25-fold	6

Supplementary Table S3. Aptamers for VEGF, distinguished into classes A through C. Bold indicates selected class representative. Our selections of aptamers chosen as representative of each of the 3 classes prioritized smaller aptamers, with higher affinity. For class A, Potty et al. (2009) carried out optimization efforts on 33t (initially derived by Gold and Janjic [28]) and determined a version with extensions at both termini which bound with better affinity[27], therefore +5′GC+3C′ was picked as the class A representative. For class B, Nonaka et al. (2013) revisited their truncated aptamer V7T1[24], and compared it with various mutant versions, leading them to a variant with better affinity termed 3R02[29]. Thus 3R02 was picked as the class B representative. For class C, Kaur et al. (2012) carried out optimization efforts on VEa5 (initially derived by Hasegawa et al.[24]) and identified a truncated version SL-2B that binds with better affinity[26], thus SL2-B was picked as the class C representative.

Class	Aptamer	Sequence	Ref**.**
A	33t	CCCGTCTTCCAGACAAGAGTGCAGGG	28
	33t-tuncated	CCGTCTTCCAGACAAGAGTGCAGGG	27
	**+5′GC+3C′**	GCCCGTCTTCCAGACAAGAGTGCAGGGC	27
B	Vap7	ATACCAGTCTATTCAATTGCACTCTGTGGGGGTGGACGGGCCGGGTAGATAGTATGTGCAATC	24
	V7T1	TGTGGGGGTGGACGGGCCGGGTAGA	24,76
	**3R02**	TGTGGGGGTGGACTGGGTGGGTACC	29
C	VEa 5	ATACCAGTCTATTCAATTGGGCCCGTCCGTATGGTGGGTGTGCTGGCCAGATAGTATGTGCAATCA	25
	Del 5	CCAGTCTATTCAATTGGGCCCGTCCGTATGGTGGGTGTGCTGGCCAG	25
	Del 5-1	ATACCAGTCTATTCAATTGGGCCCGTCCGTATGGTGGGTGTGCTGGCCAG	25
	SL12	ATACCAGTCTATTCAATTGGGCCCGTCCGTATGGTGGG	26
	**SL2-B**	CAATTGGGCCCGTCCGTATGGTGGGT	26
	VEap-twj	CTGGCCAGATACCAGTCTATTCAATTGGGCCCGTCCGTATGGTGGGTGTGCTGGCCAG	30
	2G19	CTGGCCAGGTACCAAAAGATGATCTTGGGCCCGTCCGAATGGTGGGTGTTCTGGCCAG	30

Supplementary Table S4. The sequences of all DNA aptamers tested in this work. Fluorescein label indicated by F. Sequence of the first aptamer in each dimer is indicated by an underline.

**Name**	**Sequences** (5′ to 3′)
fA	F-GCCCGTCTTC CAGACAAGAG TGCAGGGC
fB	F-TGTGGGGGTG GACTGGGTGG GTACC
fC	F-CAATTGGGCC CGTCCGTATG GTGGGT
nfAT_{100}	GCCCGTCTTC CAGACAAGAG TGCAGGGCTT TTTTTTTTTT TTTTTTTTTT TTTTTTTTTT TTTTTTTTTT TTTTTTTTTT TTTTTTTTTT TTTTTTTTTT TTTTTTTTTT TTTTTTTTTT TTTTTTTT
nfCT_{100}	CAATTGGGCC CGTCCGTATG GTGGGTTTTT TTTTTTTTTT TTTTTTTTTT TTTTTTTTTT TTTTTTTTTT TTTTTTTTTT TTTTTTTTTT TTTTTTTTTT TTTTTTTTTT TTTTTTTTTT TTTTTT
$AT_{0}C$	F-GCCCGTCTTC CAGACAAGAG TGCAGGGCCA ATTGGGCCCG TCCGTATGGT GGGT
$AT_{10}C$	F-GCCCGTCTTC CAGACAAGAG TGCAGGGCTT TTTTTTTTCA ATTGGGCCCG TCCGTATGGT GGGT
$AT_{20}C$	F-GCCCGTCTTC CAGACAAGAG TGCAGGGCTT TTTTTTTTTT TTTTTTTTCA ATTGGGCCCG TCCGTATGGT GGGT
$AT_{30}C$	F-GCCCGTCTTC CAGACAAGAG TGCAGGGCTT TTTTTTTTTT TTTTTTTTTT TTTTTTTTCA ATTGGGCCCG TCCGTATGGT GGGT
$AT_{60}C$	F-GCCCGTCTTC CAGACAAGAG TGCAGGGCTT TTTTTTTTTT TTTTTTTTTT TTTTTTTTTT TTTTTTTTTT TTTTTTTTTT TTTTTTTTCA ATTGGGCCCG TCCGTATGGT GGGT
$CT_{0}A$	F-CAATTGGGCC CGTCCGTATG GTGGGTGCCC GTCTTCCAGA CAAGAGTGCA GGGC
$CT_{10}A$	F-CAATTGGGCC CGTCCGTATG GTGGGTTTTT TTTTTTGCCC GTCTTCCAGA CAAGAGTGCA GGGC
$CT_{20}A$	F-CAATTGGGCC CGTCCGTATG GTGGGTTTTT TTTTTTTTTT TTTTTTGCCC GTCTTCCAGA CAAGAGTGCA GGGC
$CT_{30}A$	F-CAATTGGGCC CGTCCGTATG GTGGGTTTTT TTTTTTTTTT TTTTTTTTTT TTTTTTGCCC GTCTTCCAGA CAAGAGTGCA GGGC
$BT_{0}A$	F-TGTGGGGGTG GACTGGGTGG GTACCGCCCG TCTTCCAGAC AAGAGTGCAG GGC
$BT_{10}A$	F-TGTGGGGGTG GACTGGGTGG GTACCTTTTT TTTTTGCCCG TCTTCCAGAC AAGAGTGCAG GGC
$CT_{0}B$	F-CAATTGGGCC CGTCCGTATG GTGGGTTGTG GGGGTGGACT GGGTGGGTAC C
$CT_{10}B$	F-CAATTGGGCC CGTCCGTATG GTGGGTTTTT TTTTTTTGTG GGGGTGGACT GGGTGGGTAC C
$AT_{0}A$	F-GCCCGTCTTC CAGACAAGAG TGCAGGGCGC CCGTCTTCCA GACAAGAGTG CAGGGC
$AT_{10}A$	F-GCCCGTCTTC CAGACAAGAG TGCAGGGCTT TTTTTTTTGC CCGTCTTCCA GACAAGAGTG CAGGGC
$AT_{20}A$	F-GCCCGTCTTC CAGACAAGAG TGCAGGGCTT TTTTTTTTTT TTTTTTTTGC CCGTCTTCCA GACAAGAGTG CAGGGC
$AT_{30}A$	F-GCCCGTCTTC CAGACAAGAG TGCAGGGCTT TTTTTTTTTT TTTTTTTTTT TTTTTTTTGC CCGTCTTCCA GACAAGAGTG CAGGGC
$AT_{60}A$	F-GCCCGTCTTC CAGACAAGAG TGCAGGGCTT TTTTTTTTTT TTTTTTTTTT TTTTTTTTTT TTTTTTTTTT TTTTTTTTTT TTTTTTTTGC CCGTCTTCCA GACAAGAGTG CAGGGC
$CT_{0}C$	F-CAATTGGGCC CGTCCGTATG GTGGGTCAAT TGGGCCCGTC CGTATGGTGG GT
$CT_{10}C$	F-CAATTGGGCC CGTCCGTATG GTGGGTTTTT TTTTTTCAAT TGGGCCCGTC CGTATGGTGG GT
$CT_{20}C$	F-CAATTGGGCC CGTCCGTATG GTGGGTTTTT TTTTTTTTTT TTTTTTCAAT TGGGCCCGTC CGTATGGTGG GT
$CT_{30}C$	F-CAATTGGGCC CGTCCGTATG GTGGGTTTTT TTTTTTTTTT TTTTTTTTTT TTTTTTCAAT TGGGCCCGTC CGTATGGTGG GT
$CT_{60}C$	F-CAATTGGGCC CGTCCGTATG GTGGGTTTTT TTTTTTTTTT TTTTTTTTTT TTTTTTTTTT TTTTTTTTTT TTTTTTTTTT TTTTTTCAAT TGGGCCCGTC CGTATGGTGG GT
$AT_{20}A_{m}$	F-GCCCGTCTTC CAGACAAGAG TGCAGGGCTT TTTTTTTTTT TTTTTTTTAG GTCGACGGTC ACGCGACTGC GCATCA

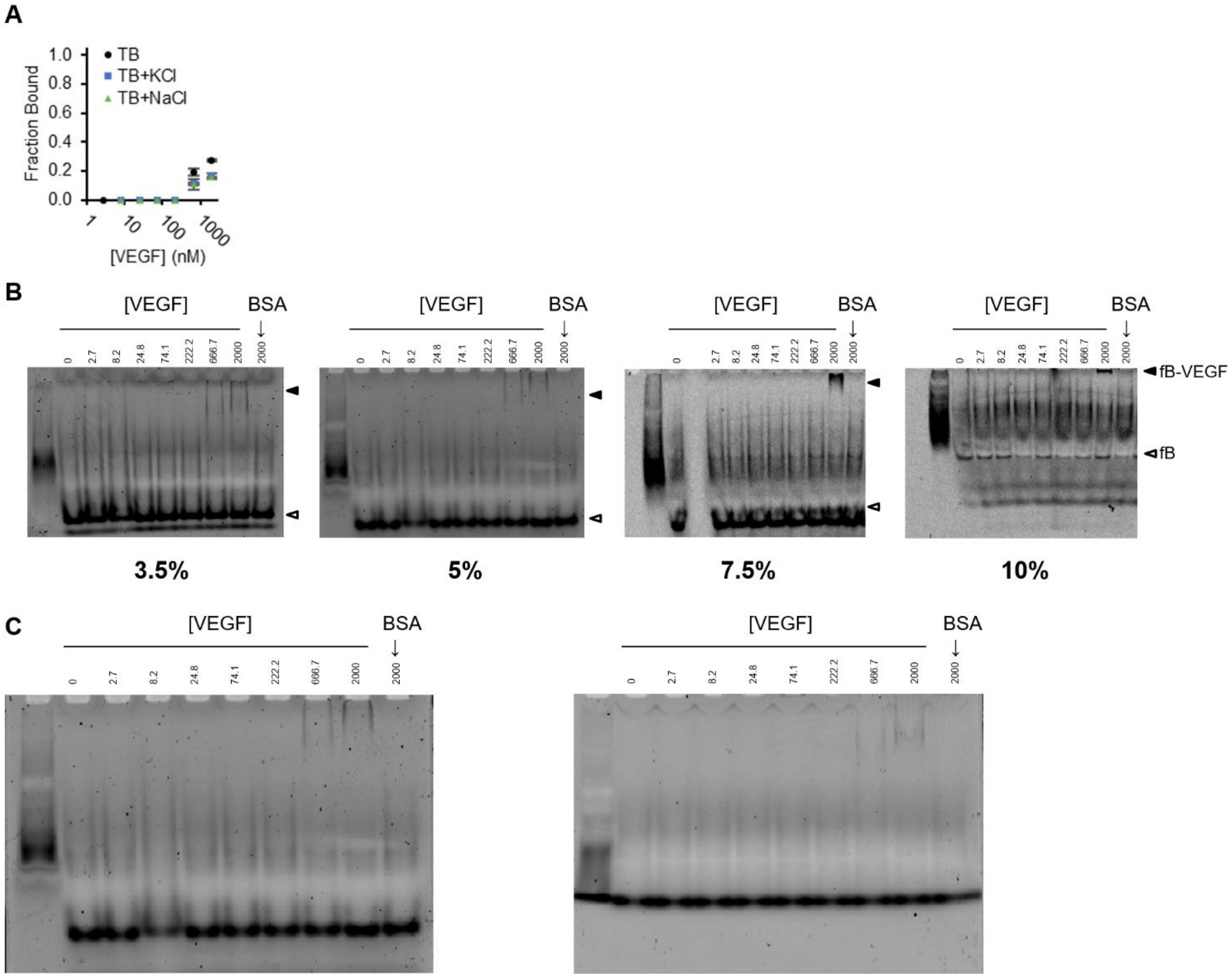

Supplementary Figure S1. Assessment of the conditions tested for optimization of EMSA for aptamer B. (**A**) Binding curves of fC with VEGF-165 in the presence of three running buffers: 0.5x TB (Tris-Borate: 44.5 mM Tris, 44.5 mM Borate, pH 8.3), 0.5x TB + KCl (50 mM), and 0.5x TB + NaCl (150 mM). (**B**) EMSA results with gels made of 3.5, 5, 7.5, 10% acrylamide. The running buffer was 0.5x TB; the gel was made of 0.5x TB. (**C**) EMSA results made with 5% acrylamide gels composed of different buffers: 0.5x TB (left gel) or PBS + 50 mM KCl. The running buffer was 0.5x TB. For each panel, fB = 2.5 nM.

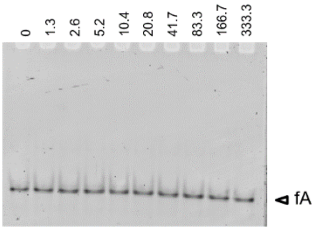

Supplementary Figure S2. Assessment of binding of fA with VEGF-121. 2.5 nM fA was mixed with 1.3, 2.6, 5.2, 10.4, 20.8, 41.7, 83.3, 166.7, and 333.3 nM VEGF-121.

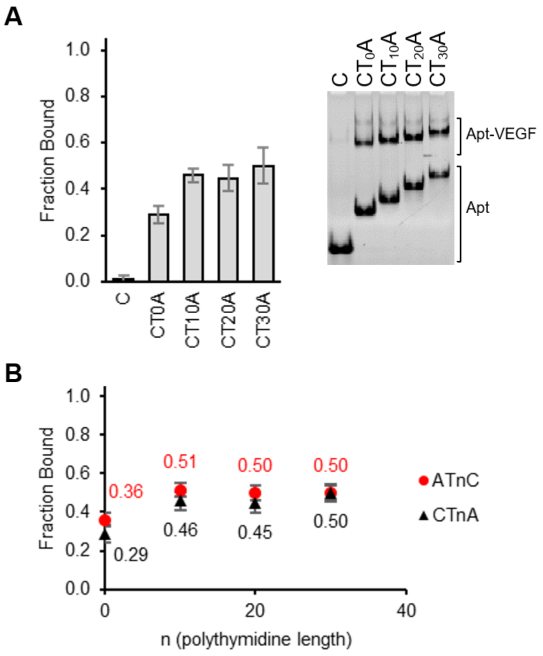

Supplementary Figure S3. Assessment of linking orientation (5′ to 3′) for heterodimers of aptamers A and C. (**A**) Fraction of fluorescent aptamers (2.5 nM) bound with VEGF-165 (10 nM). Aptamer C (lane 1) and four CT_nA aptamers were compared in this experiment (n = 0, 10, 20, 30; lanes 2-5). (**B**) Fraction of fluorescent AT_nC (red circle) and CT_nA (black triangle) heterodimers (2.5 nM) bound with VEGF-165 (10 nM).

.

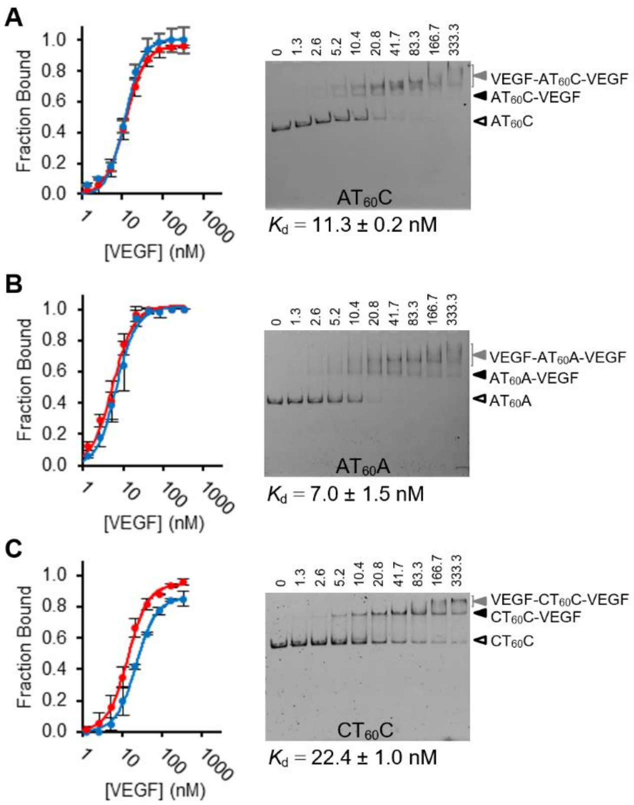

Supplementary Figure S4. Dimers with extended linkers compared. (**A**) $AT_{10}C$ (red) compared to $AT_{60}C$ (blue), with representative gel image of $AT_{60}C$ shown in right panel (**B**) $AT_{20}A$ (red) compared to $AT_{60}A$ (blue) (**C**) CT_0C (red) compared to $CT_{60}C$ (blue).

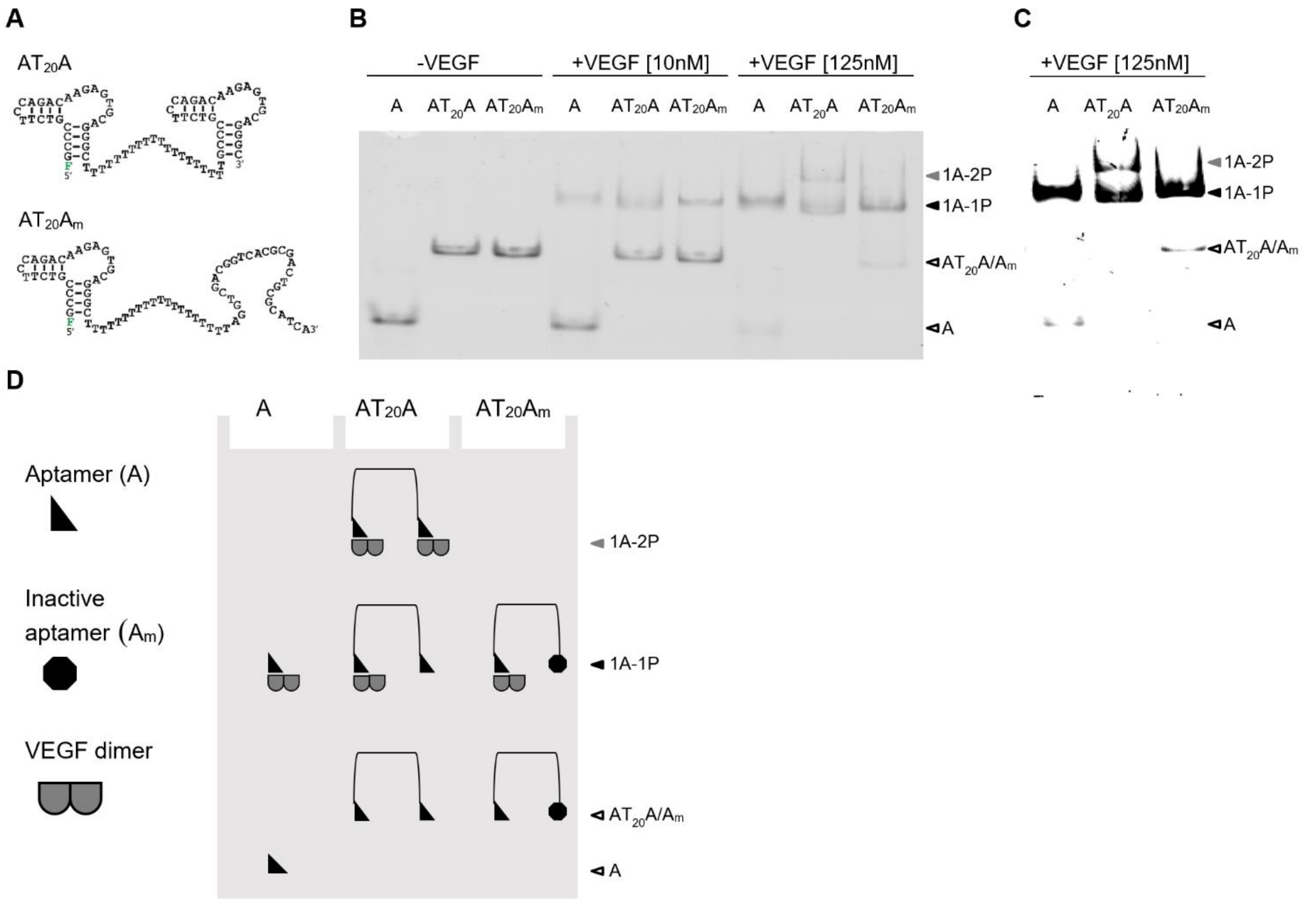

Supplementary Figure S5. Deactivation of 1:2 binding using a dimeric aptamer containing a deactivated aptamer domain. (**A**) Secondary structure of fully functional dimeric aptamer AT$_{20}$A and partially functional dimeric aptamer AT$_{20}$A$_m$ in which the sequence of the second aptamer domain is scrambled. (**B**) Binding of 2.5 nM monomeric aptamer A, AT$_{20}$A, and AT$_{20}$A$_m$ with 0, 10, and 125 nM VEGF. Unbound aptamers, 1A-1P complex (A: aptamer; P: protein), and 1A-2P complex are indicated by unfilled, black and grey arrows, respectively. (**C**) Lanes 7-9 from panel B, shown with increased intensity to demonstrate lack of 1A-2P complex with AT$_{20}$A$_m$. (**D**) Schematic explanation of what happened between the relevant aptamer and VEGF in lanes 7-9 in panel B.

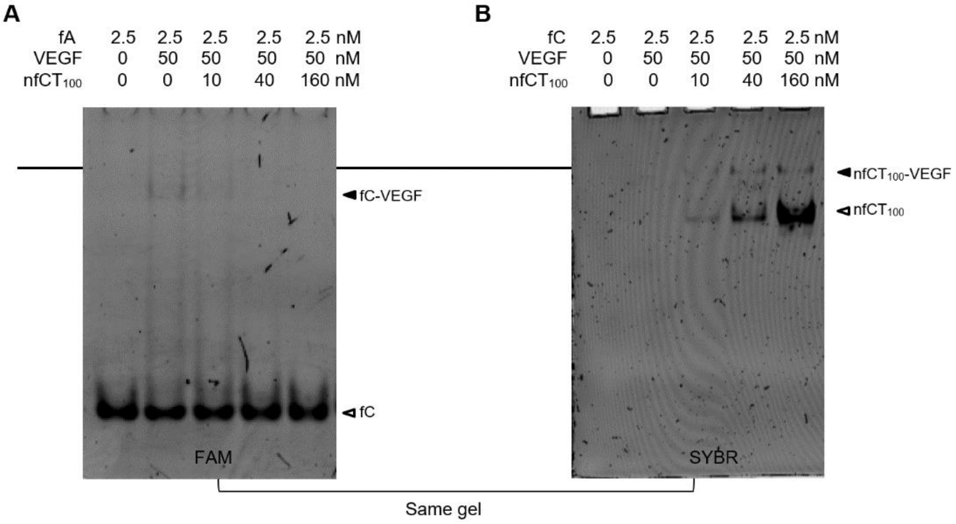

Supplementary Figure S6. fC and $nfCT_{100}$ competition assay. (**A**) Fluorescent gel image of 2.5 nM fC alone (lane 1), and in the presence of 50 nM VEGF-165 (lanes 2-5) as well as 10, 40, 160 nM (lanes 3-5, respectively) $nfCT_{100}$. (**B**) SYBR gold stained version of same gel. Black line indicates location of the $nfCT_{100}$-VEGF complex across both images of the same gel.

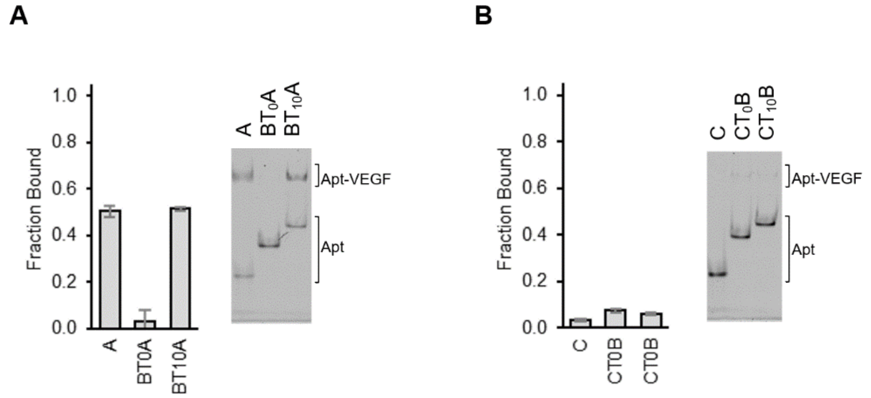

Supplementary Figure S7. Assessment of binding of heterodimeric A/B and B/C aptamers. Comparative binding of (**A**) aptamer A and two BT_nA heterodimers; (**B**) aptamer C and two CT_nB heterodimers. Fraction of each fluorescent aptamer (2.5 nM) bound with VEGF-165 (10 nM) was shown.

A

1 2 3 4 5 6 7 8 9 10

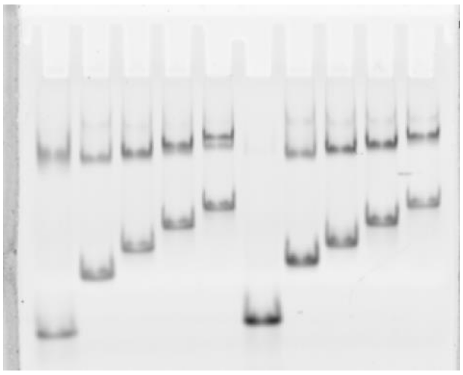

B

1 2 3 4 5 6 7 8 9 10 1 2 3 4 5 6 7 8 9 10

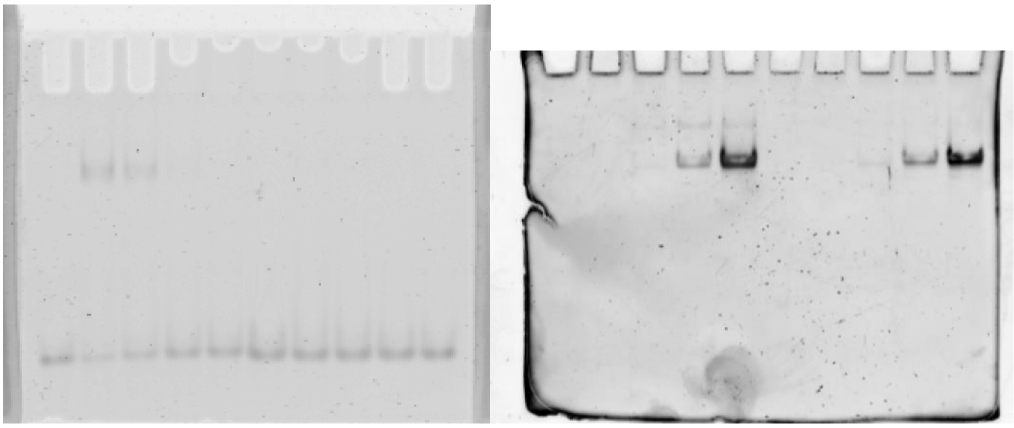

C

1 2 3 4 5 6 7 8 9 10 1 2 3 4 5 6 7 8 9 10

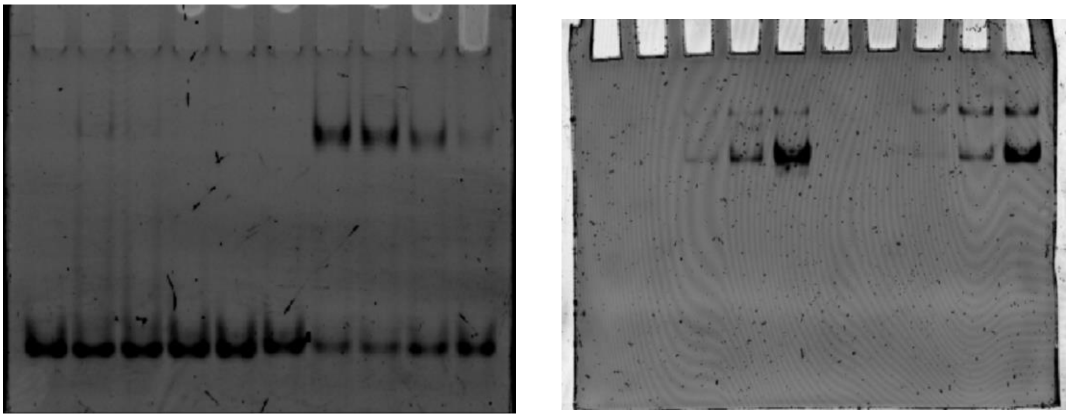

D

1 2 3 4 5 **6** **7** **8** 9 10

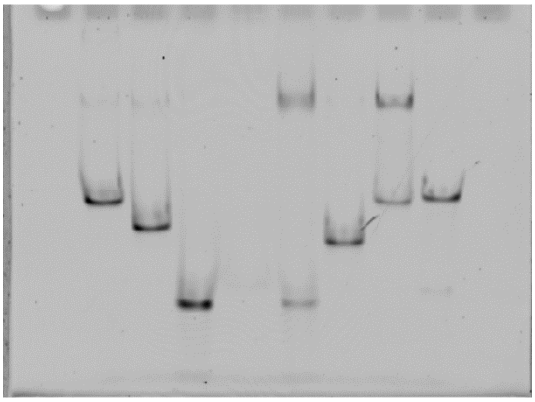

E

1 2 3 4 5 6 **7** **8** **9** 10

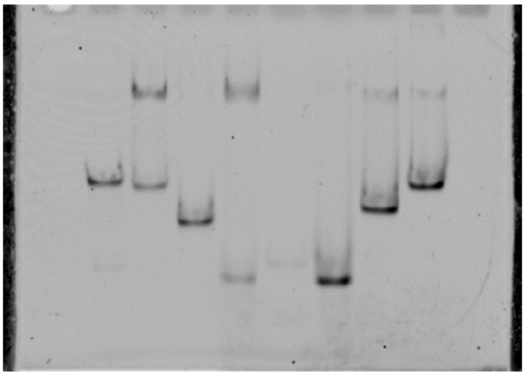

Supplementary Figure S8. (**A**) Full gel of image used in Figure 3A which showed only lanes 1-5 of this 10-well gel. Lanes 5-10 are shown in Supplementary Figure S3A. (**B**) Full gel of image used in Figure 5 which showed only lanes 1-5 of this 10-well gel; the left panel shows the gel in its glass covering, with fluorescently-labelled DNA visible, and the right panel shows the gel after staining with SYBR gold.(**C**) Full gel of image used in Figure S6 which showed only lanes 1-5 of this 10-well gel; the left panel shows the gel in its glass covering, with fluorescently-labelled DNA visible, and the right panel shows the gel after staining with SYBR gold. (**D**) Full gel of image used in Supplementary Figure S7A which showed only lanes 6-8 of this 10-well gel (**E**) Full gel of image used in Supplementary Figure S7B which showed only lanes 7-9 of this 10-well gel/

Integrated DNA Technologies

Page 1/1

Analytical ESI-MS Report

Sales Order: 14799290
Reference ID: 186839828
Manufacturing ID: 233611935

Instrument: MS-IALTQ-12
Acquired: 1/3/2018 5:48 PM
Operator ID: 3539
Reviewed: 1/3/2018 6:08 PM

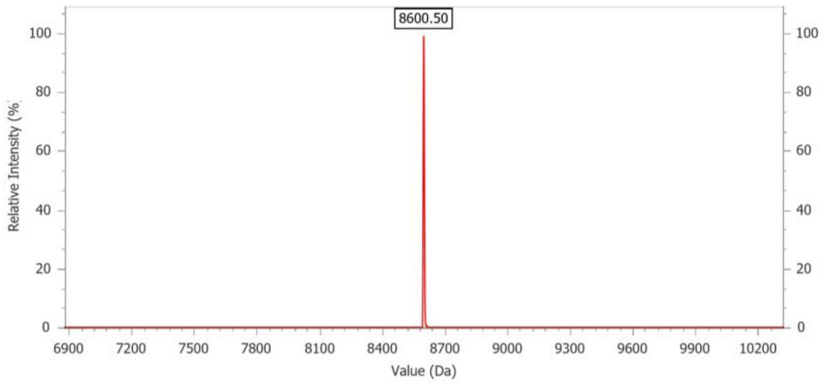

Sequence Name: A
Sequence: 5'- GCC CGT CTT CCA GAC AAG AGT GCA GGG C -3'
Calculated Molecular Weight: 8599.6
Measured Molecular Weight: 8600.50

Supplementary Figure S9. Representative image of MS data received from Integrated DNA Technologies (Coralville, IA, USA) from mass spectroscopy analysis of each oligonucleotide.
